# Exposure to acoustic stimuli promotes the development and differentiation of neural stem cells from the cochlear nuclei through the clusterin pathway

**DOI:** 10.3892/ijmm.2015.2075

**Published:** 2015-01-21

**Authors:** TAO XUE, LI WEI, DING-JUN ZHA, LI QIAO, LIAN-JUN LU, FU-QUAN CHEN, JIAN-HUA QIU

**Affiliations:** 1 Departments of Otolaryngology, The Fourth Military Medical University, Xi’an, Shaanxi 710032, P.R. China; 2 Obstetrics and Gynecology, Xijing Hospital, The Fourth Military Medical University, Xi’an, Shaanxi 710032, P.R. China

**Keywords:** neural stem cells from the cochlear nuclei, acoustic stimuli, RNA interference, clusterin, differentiation

## Abstract

Stem cell therapy has attracted widespread attention for a number of diseases. Recently, neural stem cells (NSCs) from the cochlear nuclei have been identified, indicating a potential direction for the treatment of sensorineural hearing loss. Acoustic stimuli play an important role in the development of the auditory system. In this study, we aimed to determine whether acoustic stimuli induce NSC development and differentiation through the upregulation of clusterin (CLU) in NSCs isolated from the cochlear nuclei. To further clarify the underlying mechanisms involved in the development and differentiation of NSCs exposed to acoustic stimuli, we successfully constructed animal models in which was CLU silenced by an intraperitoneal injection of shRNA targeting CLI. As expected, the NSCs from rats treated with LV-CLU shRNA exhibited a lower proliferation ratio when exposed to an augmented acoustic environment (AAE). Furthermore, the inhibition of cell apoptosis induced by exposure to AAE was abrogated after silencing the expression of the CLU gene. During the differentiation of acoustic stimuli-exposed stem cells into neurons, the number of astrocytes was significantly reduced, as evidenced by the expression of the cell markers, microtubule associated protein-2 (MAP-2) and glial fibrillary acidic protein (GFAP), which was markedly inhibited when the CLU gene was silenced. Our results indicate that acoustic stimuli may induce the development and differentiation of NSCs from the cochlear nucleus mainly through the CLU pathway. Our study suggests that CLU may be a novel target for the treatment of sensorineural hearing loss.

## Introduction

Sensorineural hearing loss is the most common sensory disorder worldwide, with approximately 300 million individuals being affected. Current clinical treatments include different types of hearing aids and cochlear implants. The number of remaining auditory neurons in patients plays a critical role in determining the effectiveness of hearing implant devices. Increasing the number of residual auditory neurons and improving the function of hearing implant devices have become key issues for optimizing the therapeutic effects of treatments for sensorineural hearing loss.

Acoustic stimuli play an important role in the development of the auditory system. The effects of exposure of the cochlea and the anterior ventral cochlear nucleus (AVCN) of C57BL/6J (B6) mice to an augmented acoustic environment (AAE) were previously reported in the study by Willott and Bross (1). Their study demonstrated that exposure to an AAE resulted in the reduced severity of the progressive loss of outer hair cells. Moreover, exposure to an AAE has been shown to exert central neuroprotective effects in DBA/2J mice, as the mice exposed to an AAE exhibited a lower elevation of auditory brainstem response thresholds, fewer missing hair cells and a markedly reduced loss of AVCN volume and neuron number compared to the untreated control mice (1,2). This previous study demonstrated that exposure to an AAE improves sensorineural hearing loss in B6 and DBA/2J mice. However, the underlying mechanisms involved in the sound-induced response of the development of the auditory system remain unclear.

Over the past decade, treatments for various diseases based on stem cells have received increasing attention due to their extensive differentiation ability (3). Accumulating evidence has confirmed that stem cells can differentiate into many types of cells and may thus be used to treat a variety of diseases, including genetic diseases, injuries, cancer, or inflammation caused by trauma (4,5,15). Based on the capacity of self-renewal and multipotency to neurons, astrocytes and oligodendrocytes, neural stem cells (NSCs) have been applied in the treatment of certain types of nervous system disorders, such as Parkinson’s disease and Alzheimer’s disease, as well as spinal cord injury and many other diseases (6–9). The first stem cells in the inner ear were found in 2003 (10). Our recent preliminary results also demonstrate that NSCs from the cochlear nuclei exist in the rat brainstem, indicating a potential treatment for sensorineural hearing loss (11).

Previous studies have found that augmented acoustic stimuli are helpful for transplanting neuronal precursor cells into the acoustic nerves (12,13). A previous study confirmed that neuronal precursor cells derived from rat olfactory bulbs can differentiate into neurons in the cochlear nuclei following exposure to an AAE (12). However, whether exposure to an AAE can affect the development of the auditory system by influencing the development of stem cells in the cochlear nuclei remains to be determined.

In the present study, we aimed to determine whether the proliferation and differentiation of NSCs exposed to acoustic stimuli could be achieved in rats. Furthermore, we investigated the mechanisms involved in the development and differentiation of acoustic stimuli-exposed NSCs in the rat cochlear nuclei.

## Materials and methods

### Animal groups

Newborn Sprague-Dawley rats (n=6) were purchased from the Animal Center at the Fourth Military Medical University, Xi’an, China. All animal procedures were performed in accordance with the National Institutes of Health guidelines, and were approved by the Institutional Animal Care and Use Committee of the Fourth Military Medical University.

The animals were randomly allocated to the following 3 groups: i) the control group with no exposure to noise; ii) the group exposed to 70 dB sound pressure level (SPL; 8 h/day); and iii) group exposed to 20 dB SPL (8 h/day). All the rats were kept in a ventilated chamber with free access to food and water.

### Isolation, culture and differentiation of NSCs from cochlear nuclei

NSCs were isolated from the cochlear nuclei of rats at 7 and 12 days after birth as previously described (1,13). The cells were incubated with Dulbecco’s modified Eagle’s medium (DMEM)/F12 medium containing B27 and N2, epidermal growth factor (EGF; 20 ng/ml) and fibroblast growth factor (FGF)-2 (20 ng/ml) and penicillin (100 U/ml) (all from Sigma-Aldrich, St. Louis, MO, USA). All cells were maintained at 37°C in a 5% CO_2_ atmosphere.

For cell differentiation, P7 cell spheres were transferred into 6-well dishes with poly-l-lysine-treated coverslips. The culture medium containing 10% fetal bovine serum (FBS), EGF and FGF-2 was removed. Half of the medium was replaced every second day. Cell differentiation was analyzed after 7 days.

### Lentiviral-mediated clusterin (CLU) shRNA delivery

LV-CLU shRNA transfection was performed in order to evaluate the effect of CLU on the development and differentiation of NSEs exposed to acoustic stimuli. For the lentiviral-mediated CLU shRNA delivery, shRNAs were designed, chemically synthesized and PAGE-purified, free of RNase contamination, according to the instructions of the manufacturer (GeneChem, Shanghai, China). They were then ligated into the pSuppres-sorNeo plasmid (provided by Dr J.S. Yan, Department of Microbiology, the Fourth Military Medical University) as previously described. Scramble oligonucleotides were used as a negative control.

Transfection was performed using Lipofectamine 2000 according to the manufacturer’s instructions as follows: 20 *μ*g of lentiviral vector carrying shRNA and 100 *μ*l Lipofectamine 2000 (Invitrogen, Carlsbad, CA, USA) were mixed and incubated with HEK293T cells (obtained from the Chinese Academy of Medeical Science, Beijing, China) at 37°C, 5% CO_2_ for 48 h. Cell supernatants were collected and concentrated using a 0.45 *μ*m filter (Amicon Ultra-15 100K; Millipore, Billerica, MA, USA); the recombinant virus was stored at −80°C until use. Newborn rats were administered an intraperitoneal injection of 2.5 *μ*g pSuppressorNeo in 1 ml of DMEM. The treated rats were exposed to environmental noise as described above. The effects of transfection were confirmed by RT-qPCR and western blot analysis.

### Immunofluorescence and fluorescence microscopy

The cells were washed 3 times with phosphate-buffered saline (PBS) and fixed with 4% formalin (Sigma-Aldrich) for 30 min at room temperature. After further washing th3 ee times with PBS containing 0.1% Triton X-100 (Sigma-Aldrich), the cells were incubated with 1% BSA for 30 min at room temperature. The cells were then cultured with anti-CLU (1:100) antibodies (Santa Cruz Biotechnology, Inc., Santa Cruz, CA, USA) and dissolved overnight at 4°C in PBS. After washing, FITC-conjugated goat anti-rabbit IgG (Sigma-Aldrich) was added at a dilution of 1:50. Observations were performed immediately after washing with fresh medium under an Olympus IX70 microscope (Olympus, Tokyo, Japan).

### Reverse transcription-quantitative (real-time) polymerase chain reaction (RT-qPCR)

Total cellular RNA was isolated from the cells using purification mini kits (RNeasy; Qiagen, Hilden, Germany) according to the manufacturer’s specifications. One microgram of total RNA was converted to cDNA (iScript cDNA Synthesis kit; Bio-Rad, Hercules, CA, USA) and was subsequently subjected to RT-PCR. PCR was performed in a thermal cycler at 94°C for 5 min followed by 30 amplification cycles (94°C, 30 sec; 55°C, 30 sec; 72°C, 1 min). Quantitative PCR was carried out to quantify the expression of CLU, Nestin, microtubule-associated protein 2 (MAP-2), glial fibrillary acidic protein (GFAP), myelin basic protein (MBP), and β-actin messenger RNA on an ABI 7300 PCR machine (Applied Biosystems, Foster City, CA, USA). The relative expression value of the target gene was calculated as the ratio of target cDNA to β-actin. The primers used in this study are presented in Table I.

### Western blot analysis

The proteins were prepared by suspending the cells in lysis buffer with complete ethylenediaminetetraacetic acid (EDTA)-free protease-inhibitor-cocktail tablets (Roche, Basel, Switzerland) for 10 min on ice. The cell extracts were sonicated (3x10 sec), then centrifuged at 15,000 × g for 15 min to remove the insoluble materials.

Each protein sample was electrophoresed by 12% sodium dodecyl sulfate-polyacrylamide gel electrophoresis (SDS-PAGE), and then transferred onto nitrocellulose membranes (Millipore, Bedford, MA, USA). After blocking with 5% fat-free milk powder at room temperature for 2 h, the membranes were incubated with primary antibodies against CLU (sc-8354), Nestin (sc-33677), Musashi RNA-binding protein 1 (Msi-1; sc-135721), MAP-2 (sc-74421), GFAP (sc-33673), MBP (sc-13564) and β-actin (sc-8432; Santa Cruz Biotechnology, Inc.) at 4°C overnight, and subsequently cultured with HRP-conjugated goat anti-mouse IgG (sc-2489; Santa Cruz Biotechnology, Inc.) for 2 h at room temperature. β-actin was used as an internal control. The reaction product was visualized by enhanced chemiluminescence (ECL; Amersham Pharmacia, Piscataway, NJ, USA).

### Flow cytometric analysis of apoptosis

To investigate the effects of CLU on the apoptosis of NSCs exposed to acoustic stimuli, we performed flow cytometric analysis of phosphatidylserine externalization using the Annexin V-fluorescein isothio-cyanate kit (Immunotech, Marseille, France). The treated cells were harvested and washed once with ice-cold PBS, and then re-suspended in binding buffer (10 mM HEPES, pH 7.4, 150 mM NaCl, 2.5 mM CaCl_2_, 1 mM MgCl_2_, 4% bovine serum albumin). Annexin V-fluorescein isothiocyanate (0.5 lg/ml) and propidium iodide (0.6 lg/ml) were added to a 250 *μ*l aliquot (5x10^6^ cells) of this cell suspension. Following incubation in the dark at room temperature for 15 min, the stained cells were immediately analyzed using a FACSCalibur™ flow cytometer (BD Biosciences, San Jose, CA, USA).

### Cell proliferation assay

To determine the effects of CLU on cell proliferation following exposure to an AAE, cell viability was ascertained by MTT assay. Targeting cells were seeded in 200 *μ*l of growth medium in a 96-well plate and cultured at 37°C overnight in 5% CO_2_. At each suitable time point, 20 *μ*l of 5 mg/ml MTT (Sigma-Aldrich) were added. The cells were further incubated at 37°C for 4 h, and the formazan crystals were then dissolved with DMSO. The optical density was determined using a microculture plate reader (BD Biosciences) at 490 nm. The absorbance values were normalized to the values obtained from the untreated cells to determine the percentage of cell survival.

### Statistical analysis

Statistical analyses were performed using ANOVA or Student’s t-tests with SPSS software (version 13.0, SPSS Inc., Chicago, IL, USA). P-values <0.05 were considered to indicate statistically significant differences.

## Results

### Acoustic stimuli induce the development and differentiation of NSCs

**Figure 1:**
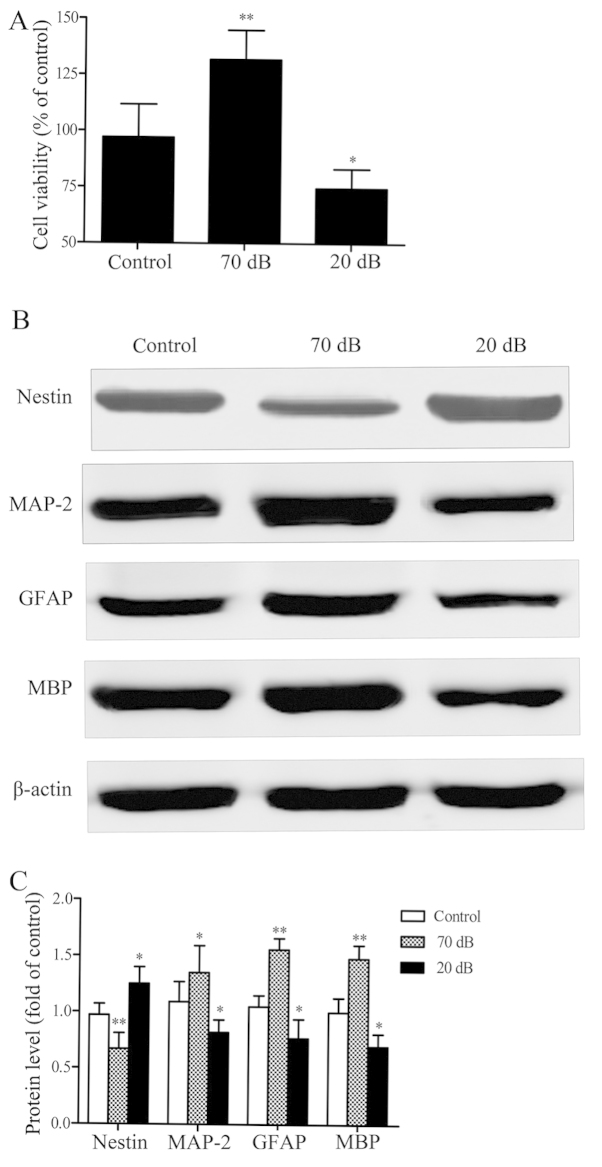
Effects of exposure to acoustic stimuli on cell development and differentiation. (A) Cell viability was estimated by MTT assay. (B) Western blot analysis of protein expression during cell differentiation. (C) Quantification of relative protein expression. In (B), β-actin served as the loading control. Results are presented as the means ± standard error; ^**^P<0.01 and ^*^P<0.05 compared to controls.

To determine the effects of acoustic stimuli on NSC proliferation and differentiation, MTT assay and western blot analysis were performed. Exposure to an AAE increased cell viability compared with the control group (Fig. 1A). Exposure to acoustic stimuli resulted in the downregulation of the NSC marker, Nestin, and the upregulation of the neuronal marker, MAP-2 (Fig. 1B and C). Exposure to acoustic stimuli also resulted in a 1.56- and 1.48-fold increase in the expression of the astrocyte marker, GFAP, and the oligodendrocyte marker, MBP, respectively.

### Exposure to different levels of acoustic stimuli induces the differential expression of CLU in NSCs

**Figure 2:**
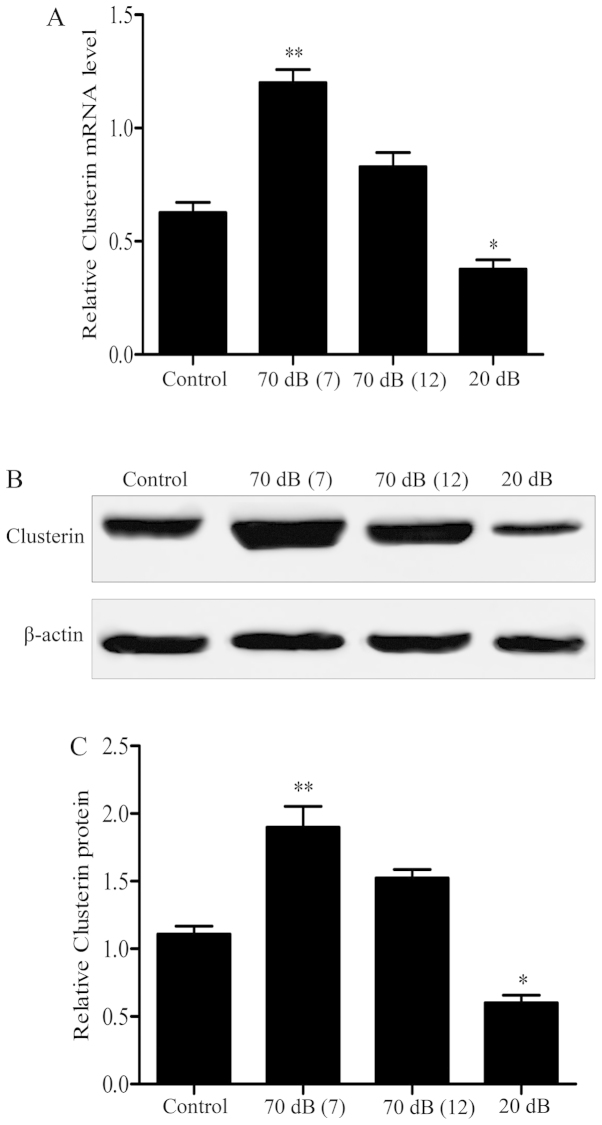
Effects of exposure to acoustic stimuli on the expression of clusterin (CLU) in neural stem cells (NSCs) from the cochlear nuclei. (A) RT-qPCR of CLU mRNA expression in NSCs and (B) western blot analysis of CLU protein expression in NSCs followig exposure to different levels of acoustic stimuli, and (C) quantification analysis of relative CLU protein expression. In (B), β-actin served as the loading control. Results are presented as the means ± standard error; ^**^P<0.01 and ^*^P<0.05 compared to controls.

To clarify the underlying mechanisms involved in the proliferation and differentiation of acoustic stimuli-exposed NSCs, the expression levels of CLU were determined. Exposure to strong acoustic stimuli (70 dB) increased the CLU mRNA levels after 7 days compared with the control group (Fig. 2A). Following exposure for 12 days, the expression levels of CLU mRNA were lower in contrast to those at 7 days, whereas the CLU mRNA levels decreased following exposure to a weakened acoustic environment (20dB).

**Figure 3:**
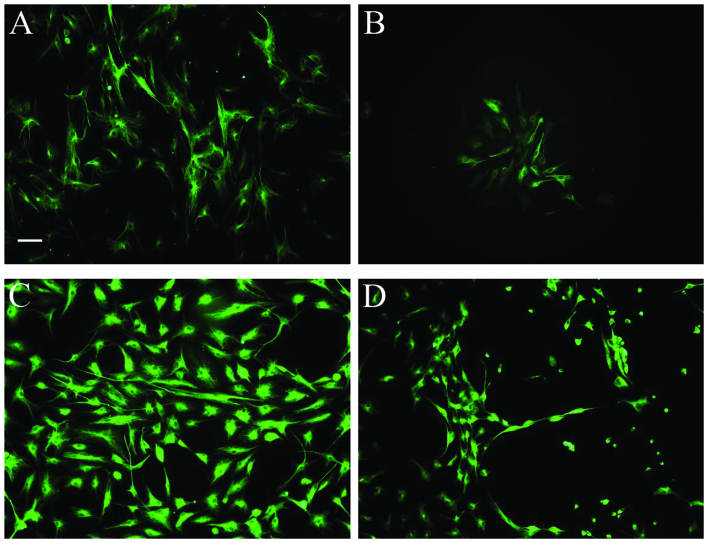
Immunofluorescence staining with antibodies against clusterin (CLU) in neural stem cells (NSCs) from the cochlear nuclei (protein expression of CLU). (A) Exposure to normal environment [about 40 dB sound pressure level (SPL)]; (B) exposure to weakened acoustic environment (20 dB SPL) for 7 days; (C) exposure to augmented acoustic environment (70 dB SPL) for 7 days; (D) exposure to augmented acoustic environment (70 dB SPL) for 12 days. Scale bar represents 50 *μ*m.

As shown by western blot analysis, the protein expression level of CLU was significantly higher in the group exposed to an AAE than in the control group. By contrast, the protein expression level of CLU in the group exposed to a weakened acoustic environment decreased. However, the CLU protein levels increased after 7 days (Fig. 2B) and exhibited higher levels of CLU than after 12 days of exposure to an AAE (Fig. 2C). Immunofluorescence staining revealed a similar effect of acoustic stimuli on CLU protein levels (Fig. 3); the protein expression of CLU increased in the group exposed to an AAE during cell differentiation, indicating a potential role of CLU in the development and differentiation of NSCs exposed to acoustic stimuli.

### Transfection with LV-CLU shRNA markedly decreases the expression of CLU in NSCs from cochlear nuclei

**Figure 4:**
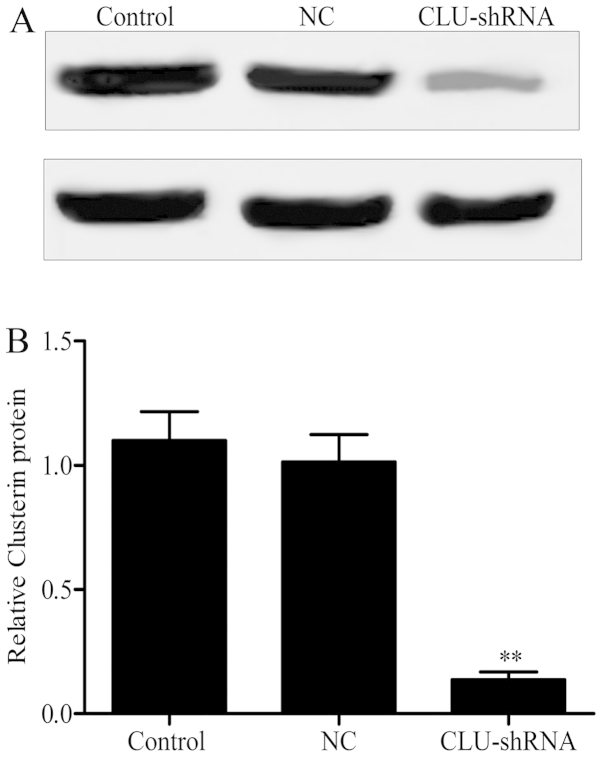
Effective silencing of clusterin (CLU) expression in rat stem cells using the specific shRNA vector: (A) western blot analysis of CLU protein expression in neural stem cells (NSCs) from the cochlear nuclei following transfection with LV-CLU shRNA, and (B) quantification of CLU protein expression. In (A), β-actin served as the loading control. Results are presented as the means ± standard error; ^**^P<0.01 compared to controls.

To determine the effects of CLU on the development and differentiation of NSCs exposed to acoustic stimuli, transfection with LV-CLU shRNA was performed. A marked downregulation in CLU protein expression was observed in the LV-CLU shRNA-treated rats compared with the controls (Fig. 4A). CLU protein levels were decreased by 83.4% in the LV-CLU shRNA-treated group (Fig. 4B). No CLU gene silencing was observed in the scramble control (negative control) group.

### Effect of CLU on NSC proliferation in rats exposed to an AAE

**Figure 5:**
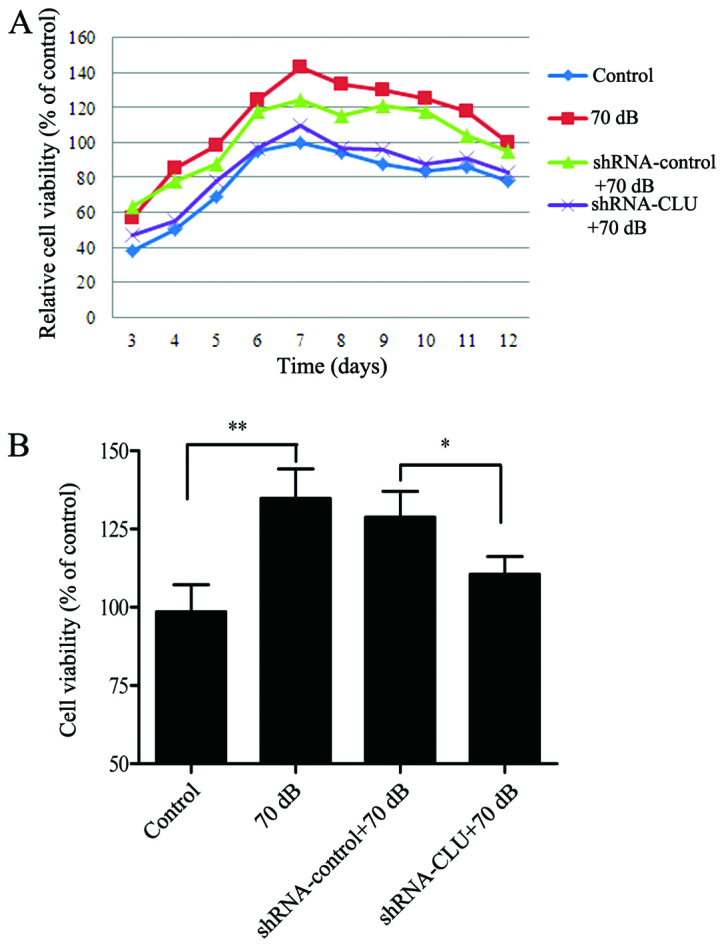
Effects of clusterin (CLU) gene silencing on cell growth. (A) Cell viability was estimated by MTT assay, and (B) quantification of relative cell viability. Results are presented as the means ± standard error; ^**^P<0.01 and ^*^P<0.05 compared to respective controls.

To determine the effects of CLU on NSC proliferation following exposure to an AAE, MTT assay was performed to determine cell viability. Cell viability was increased following exposure to AAE and reached a peak after 7 days (Fig. 5A). Following silencing of CLU expression, the AAE-induced cell proliferation decreased. Exposure to an AAE induced an increase in cell viability to approximately 131%, which was attenuated when the expression of the CLU gene was silenced (Fig. 5B). Our results suggest that CLU is involved in the proliferation of NSCs induced following exposure to acoustic stimuli.

### CLU silencing ameliorates the inhibition of cell apoptosis induced by exposure to acoustic stimuli in rat NSCs

**Figure 6:**
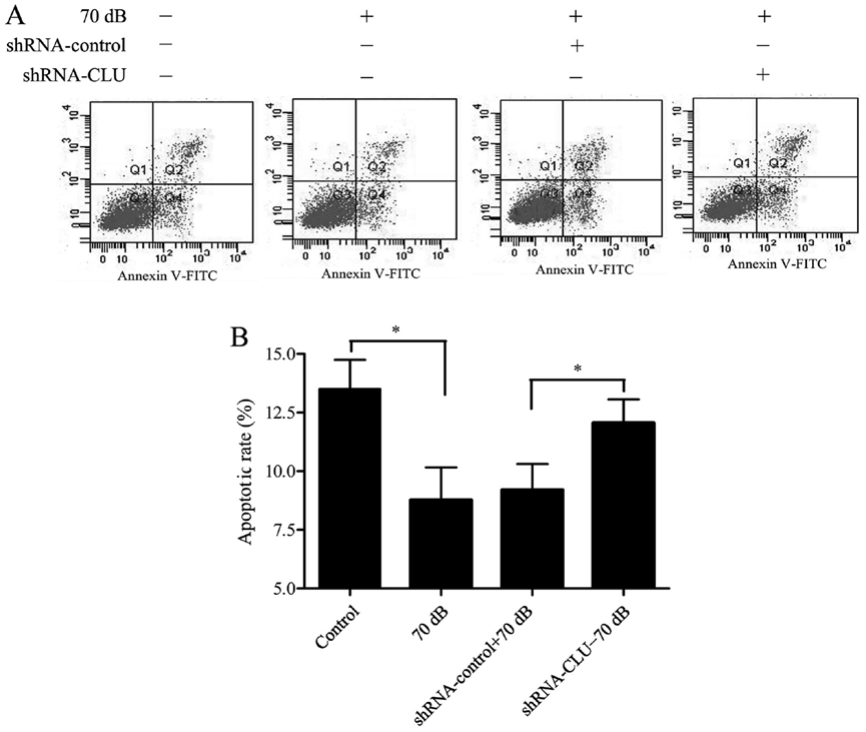
Clusterin (CLU) gene silencing through active cellular apoptosis in an augmented acoustic environment (AAE). (A) Cells were analyzed by Annexin V-fluorescein isothiocyanate, and (B) quantification of relative apoptotic cells. In (A), propidium iodide (PI) staining was conducted as described in the Material and methods section with Q2 denoting necrotic or late apoptotic cells (Annexin V1, PI1), Q3 corresponding to viable or undamaged cells (Annexin V^−^, PI^−^), and Q4 identifying cells undergoing early apoptosis (Annexin V1, PI2). Results are presented as the means ± standard error; ^*^P<0.05 compared to respective controls.

To investigate the effects of CLU on NSC apoptosis following exposure to acoustic stimuli, flow cytometric analysis of phosphatidyl-serine externalization (by fluorescence-labeled Annexin V binding) was used. Our results revealed that the inhibition of cell apoptosis following exposure to an AAE was reversed when the CLU gene was silenced. Following exposure to an AAE, the cell apoptotic rate significantly decreased, whereas the apoptotic rate increased following the silencing of the CLU gene (Fig. 6B). These results suggest that CLU is involved in the inhibition of cell apoptosis induced by exposure to acoustic stimuli.

### Role of the CLU gene during cell differentiation triggered by exposure to acoustic stimuli

**Figure 7:**
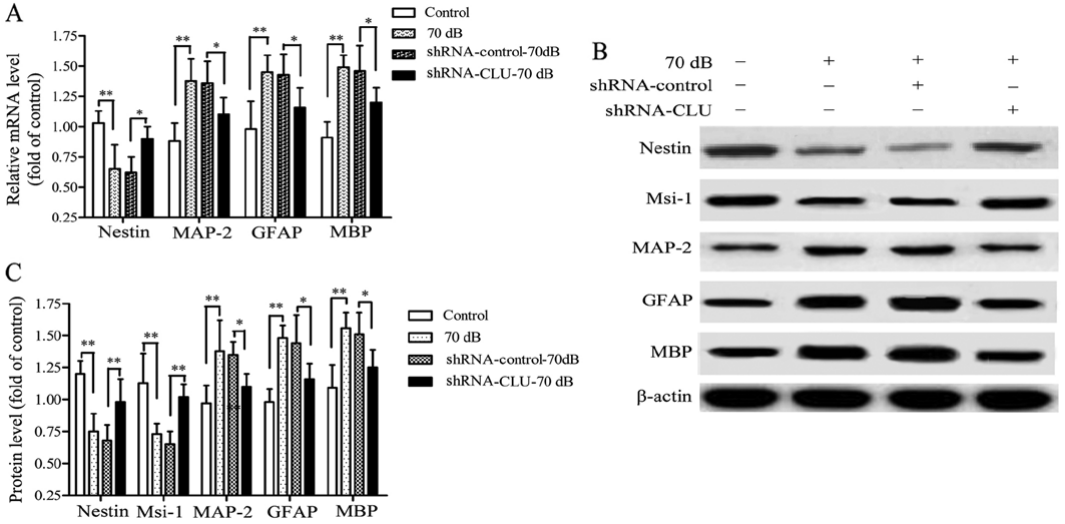
Effect of clusterin (CLU) gene silencing on cell differentiation in an augmented acoustic environment (AAE). (A) mRNA expression during cell differentiation confirmed by RT-qPCR, (B) protein expression during cell differentiation measured by western blot analysis, and (C) quantification of relative protein expression. In (A), β-actin served as the loading control. Results are presented as the means ± standard error; ^**^P<0.01 and ^*^P<0.05, compared to respective controls.

The CLU gene plays a role during cell differentiation which is triggered by acoustic stimuli. Following exposure to an AAE, the depletion of CLU led to the downregulation of the mRNA levels of the NSC markers, Nestin and Msi-1, and the upregulation of the neuronal marker, MAP-2. The formation of astrocytes and oligodendrocytes also exhibited certain changes. Exposure to acoustic stimuli resulted in an increase of 1.65- and 1.45-fold in the expression of the astrocyte marker, GFAP, and the oligo-dendrocyte marker, MBP, respectively. However, the increase in these levels was suppressed when CLU was silenced. The results from western blot analysis conferred similar changes in the protein levels of these markers. The protein levels of MAP-2, GFAP and MBP increased by 1.35-, 1.48- and 1.56-fold, respectively (Fig. 7). The increase in these levels was also suppressed when CLU was silenced. Our observations reveal that exposure to acoustic stimuli affects cell differentiation through CLU.

## Discussion

Sensorineural hearing loss is a seriously debilitating condition for millions of individuals worldwide. The dramatic increase in the incidence of hearing loss is due to the overexposure to environmental toxins, genetic predisposition to age-related hearing loss, or both. The problem is further compounded as the human population continues to age. The number of Americans who suffer substantial hearing impairment has been estimated to rise to as many as 65 million individuals by the year 2030 (14). This will undoubtedly have enormous socioeconomic impact for patients, health care providers and policymakers.

Acoustic stimuli play an important role in the development of the auditory system. It has been suggested that exposure to an AAE affects the proliferation and differentiation of NSCs derived from rat olfactory bulbs (12). At present, treatments with pluripotent stem cells have been applied in the treatment of a variety of diseases, including Parkinson’s disease and Alzheimer’s disease, as well as spinal cord injury, heart failure and bone marrow dysfunction (7–9,15). Our preliminary results indicated that NSCs exist in the rat cochlear nuclei (11). Our finding is significant for treating hearing loss by inducing the differentiation of NSCs. As expected, exposure to an AAE significantly enhanced the proliferation and differentiation of NSCs from cochlear nuclei in the rat auditory system. Exposure to an AAE may improve the treatment of sensori-neural hearing loss by regulating the development of NSCs from cochlear nuclei. However, the underlying mechanisms involved in the development of NSCs exposed to acoustic stimuli remain unclear.

CLU, which is also known as apolipoprotein J, is a highly conserved and heterodimeric-secreted glycoprotein, and is found in virtually all human tissues and fluids (16). CLU has proven to be involved in the regulation of cell proliferation and differentiation in a number of tissues (17,18). Furthermore, studies have confirmed that the CLU protein is associated with various physiological processes, such as cell adhesion, cell differentiation and transformation, cell cycle regulation, immune regulation, lipid transportation and DNA repair (19,20). It has been demonstrated that CLU promotes the growth of corneal epithelial cells through the upregulation of mesenchymal cells *in vitro* (21). To clarify the underlying mechanisms involved in the proliferation and differentiation of NSCs from cochlear nuclei exposed to acoustic stimuli, we determined the expression levels of CLU. We observed an upregulation in CLU expression during the differentiation of NSCs from cochlear nuclei exposed to an AAE. Our results confirm an important role of CLU in the development of NSCs exposed to acoustic stimuli.

Stem cells are a special class of cells that have self-renewal and pluripotent differentiation capacities (6). During heart failure, the granulocyte-colony stimulating factor induces cardiac regeneration by increasing the number of peripheral blood stem cells and transferring them to the site of injury (22,23). These data indicate that the proliferation of stem cells is crucial to stem cell therapy. Acoustic stimuli have a close relationship with newborn rat brainstem NSCs from cochlear nuclei and can promote the proliferation of NSCs from cochlear nuclei and inhibit their apoptosis. However, we found that cell proliferation decreased after CLU was silenced in rats exposed to acoustic stimuli. Acoustic stimuli appear to promote the proliferation of NSCs from the cochlear nuclei through CLU.

NSCs have been widely used in the treatment of nerve injury-related diseases, partly due to their pluripotent differentiation potential. Sugaya *et al* (24,25) demonstrated that NSCs improved cognitive function in model rats with Alzheimer’s disease. Furthermore, NSCs can differentiate into dopaminergic neurons to treat Parkinson’s disease, indicating an important role of NSC differentiation in the therapeutic effects (26). Nestin is a member of the family of intermediate filaments and is found in neuroepithelial stem cells (27). Nestin is widely used to identify NSCs as its expression is terminated when neural precursor cells differentiate into neurons and glial cells (28). Msi-1 is abundant in neural precursor and stem cells, and is known as a common marker for neural precursor cells (29). MAP-2 is primarily expressed in the nervous system and is one of the most abundant proteins in the brain (30). It is thought to be a good candidate as a biomarker for neurons. GFAP is a monomeric intermediate filament protein found in the astroglial skeleton (31). GFAP has been found to be a potentially useful astrocyte marker in predicting clinical outcomes (32). In this study, exposure to acoustic stimuli induced a decrease in the expression of the NSC markers, Nestin and Msi-1, and an increase in the expression of MAP-2, GFAP and MBP. However, these effects were markedly attenuated when the CLU gene was silenced, indicating that acoustic stimuli promotes the differentiation of NSCs from the cochlear nuclei through the CLU pathway.

In conclusion, this study confirms that exposure to acoustic stimuli promotes the proliferation and differentiation of NSCs from the cochlear nuclei through the CLU pathway. Our data support a new potential aspect for the treatment of sensorineural hearing loss.

## Figures and Tables

**Table I:** Sequence of oligonucleotides used as PCR primers.

Gene	Position	Primer sequences
CLU	Up	5′-ATGATGAAGACTCTGCTGCT-3′
	Down	5′-TCACTCCTCCCGGTGCTT-3′
Nestin	Up	5′-TCGCTTAGAGGTGCAACAGC-3′
	Down	5′-CCTATTCCGTCTCAACGTA-3′
Msi-1	Up	5′-CGAGCTCGACTCCAAAACAAT-3′
	Down	5′-AGCTTTCTTGCATTCCACCA-3′
GFAP	Up	5′-GAGATCGCCACCTACAGGAA-3′
	Down	5′-GCTCCTGCTTCGACTCCTTA-3′
MAP-2	Up	5-TCAGAGGCAATGACCTTACC-3′
	Down	5′-GTG GTA GGC TCT TGG TCT TT-3′
MBP	Up	5′-TCGCAGAGGACCCAAGATG-3′
	Down	5′-TAAGAAGCCGAGGGCAGGA-3′
β-actin	Up	5′-CAGGATGCAGAAGGAGATTAC-3′
	Down	5′-AACGCAGCTCAGTAACAGTC-3′

CLU, clusterin; Msi-1, Musashi RNA-binding protein 1; GFAP, glial fibrillary acidic protein; MAP-2, microtubule-associated protein 2; MBP, myelin basic protein.
