# [Expression of Concern] Exposure to acoustic stimuli promotes the development and differentiation of neural stem cells from the cochlear nuclei through the clusterin pathway

**DOI:** 10.3892/ijmm.2026.5933

**Published:** 2026-07-20

**Authors:** Tao Xue, Li Wei, Ding-Jun Zha, Li Qiao, Lian-Jun Lu, Fu-Quan Chen, Jian-Hua Qiu

Int J Mol Med 35: 637-644, 2015; DOI: 10.3892/ijmm.2015.2075

Following the publication of this paper, it was drawn to the Editor's attention by a concerned reader that various of the flow cytometry plots featured in Fig. 6A subsequently appeared in six papers written by different authors at different research institutes that were published in the years 2017-8, one of which has since been retracted (a paper in *Oncology Research*). Furthermore, the possible duplication of a western band for the Msi-1 experiments shown in Fig. 7B was also noted on p. 643, where the same data were apparently used to present two different experimental conditions.

The authors were contacted by the Editorial Office to offer an explanation for the issues described above, and they have replied to say that, since this article is old, the digitized raw data for this paper were discarded in accordance with the institute's data retention policy after the mandated retention period; moreover, the authors maintain full confidence in the validity and reproducibility of their overall findings. Owing to the fact that the Editorial Office has been made aware of potential issues surrounding the scientific integrity of this paper, however, we are issuing an Expression of Concern to notify readers of this potential problem.

